# From West to East: Professor Pavlos Savvidis’ Quest for Light

**DOI:** 10.1038/s41377-024-01616-w

**Published:** 2024-09-26

**Authors:** Ji Wang

**Affiliations:** Westlake University, No. 600 Dunyu Road, Xihu District, Hangzhou, Zhejiang 310030 China

**Keywords:** Optics and photonics, Lasers, LEDs and light sources

## Abstract

As early as about 2400 years ago, Mozi (original name Mo Di, Latinized as Micius), an ancient Chinese scientist, proposed the theory of pinhole imaging that demonstrates the fundamental principle of light behavior. About 700 years ago, Marco Polo, an Italian explorer, traveled to China along the Silk Road, marveled at the economic prosperity and the advanced technology of Hangzhou City in China, and described Hangzhou as “the most beautiful and splendid city in the world”. About 5 years ago, with the support of the China-proposed Belt-and-Road Initiative, it was in Hangzhou City that Professor Pavlos Savvidis, an Armenian-born Greek physicist, chose to work with more of his Chinese counterparts and took on the challenge of building a new research laboratory on quantum optoelectronics. He used to study and work in the UK, the USA, and Greece, but now in New China’s first new type of research university supported by the society—Westlake University. Traveling from West to East, traversing from one civilization to another, Professor Pavlos Savvidis delves into his unwavering quest for light in this issue of “Light People”, and discusses his tireless pursuit of excellence in the field of optoelectronics, which has garnered him widespread citation, recognition, and contribution to the global scientific community.

**Short Bio:** Pavlos Savvidis received his B.Sc. in physics from the University of Athens, Greece, and his doctoral degree from the University of Southampton, UK in 2001. From 2002 to 2004, he was a post-doctoral associate at the Department of Physics of the University of California, Santa Barbara, USA. Since 2004, he had served as Assistant Professor at the Department of Materials Science and Technology of the University of Crete, Greece, retiring in 2023 as Full Professor. In 2014, he was awarded the prestigious one-year Leverhulme Fellowship to work as a Visiting Professor at Cavendish Labs, University of Cambridge. Since 2023, he has been Full Professor at the School of Science in Westlake University, China. He has published over 110 research papers in high-impact journals such as *Nature, Science, Physical Review Letters,* etc. His papers have been cited over 7000 times. He is the inventor of the polariton amplifier and has demonstrated the concept of polariton LED at room temperature. He has served as a referee for leading physical journals, including *Nature, Physical Review Letters,* and others.
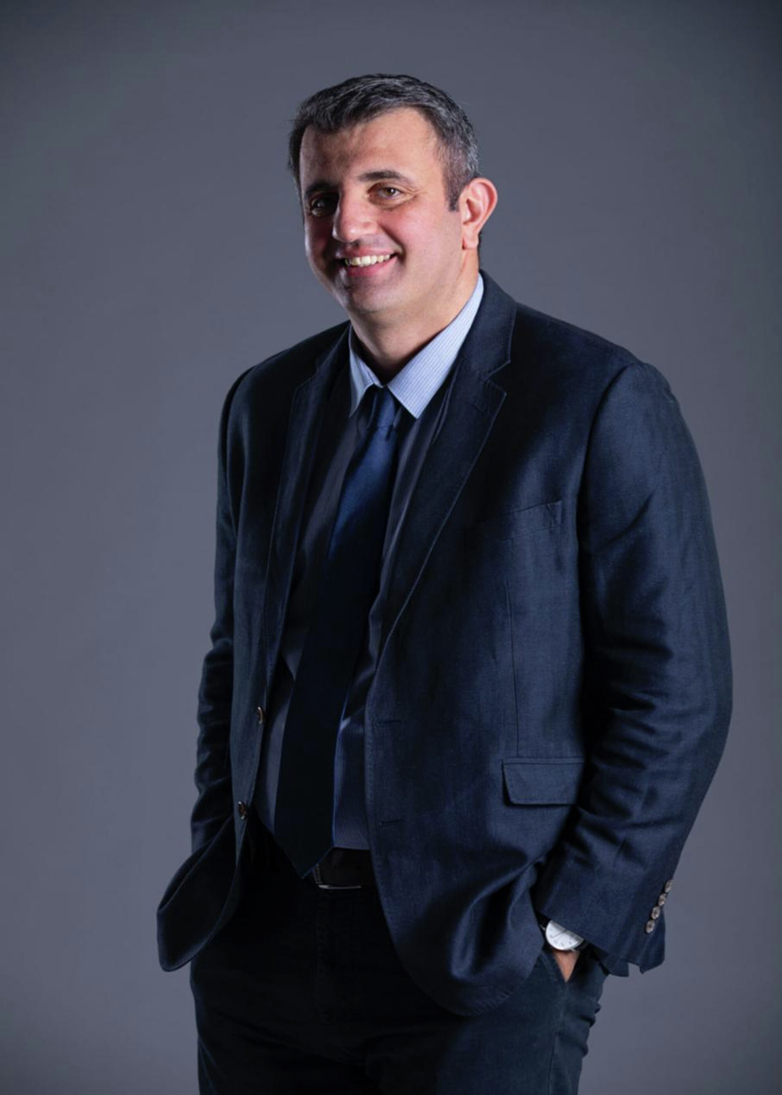


**Q1: Could you please tell me about your hometown? What has inspired you to pursue a career as a physicist? Who has had the most significant influence on your career?**

A1: I grew up and received my initial education in the city of Yerevan of the former Soviet Republic of Armenia. In fact, the whole neighborhood where I lived was built around a major physics research institute—the Yerevan Institute of Physics, hosting electron synchrotron with the main focus on elementary particle physics, in which both of my parents worked as theoretical physicists. Such a microclimate certainly had a major impact on my vision of the world and my interest in physics. During my childhood, with limited commercial toys available, my father and I used to make home-built toys exploiting principles of aerodynamics, hydrodynamics, electromagnetism or light for that matter. Such experimental activity was very creative and inspirational with physical principles at its foundations.

However, it was through the participation and winning of Physics Olympiad in Armenia that I truly discovered my interest and talent in physics. Traveling to Tashkent in Uzbekistan as a teenager to participate in the Soviet Olympiad in physics was profoundly inspirational and solidified my choice to become a physicist.

Significantly, the critical choices I made later in life have truly defined my scientific path up until today. When I was little, my father told me the story of the ancient Greek Goddess—Tyche, who is often depicted with a lock of hair hanging over her forehead, representing the fleeting nature of luck. Just as one must catch hold of Tyche by this forelock before she passes by, we must recognize and grasp opportunities swiftly when they arise. I had several such moments myself. I remember sitting at the railway station in Cambridge, UK, where I had just received a PhD offer in experimental ultrafast laser spectroscopy. At that moment, I decided to follow my instinct and pursue experimental rather than theoretical physics, a decision that significantly shaped my career. Had I chosen differently, my life would be very different today.

**Figure:**
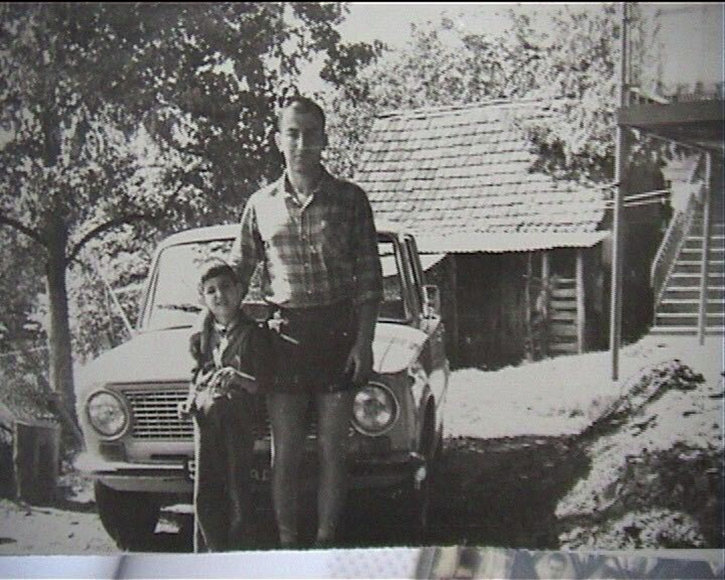
Prof. Pavlos Savvidis and his father during their summer vacation in Iraga, Georgia, 1981

**Q2: You created the first polariton amplifier during your Ph.D. in the University of Southampton. Could you explain its significance and applications?**

A2: For years, researchers around the world have been looking for the elusive evidence of Bose-Eistein condensation of excitons, namely Coulomb force bound electron-hole pairs in semiconductors. While I was starting my PhD research in 2000, there were several theoretical predictions of condensation of hybrid exciton-photon quasiparticles called polaritons. It was predicted that by dressing excitons with photons it would be possible to suppress mutual scattering between quasiparticles which could allow macroscopic condensation of polaritons at cryogenic and even elevated temperatures.

My experiments as a PhD student in the University of Southampton showed that polaritons indeed behaved as bosons accumulating in the ground state of the system via stimulated scattering, thus revealing their bosonic property. When we discovered this fundamental process, owing to its sharp angular selectivity satisfying phase matching conditions, we named the effect “magic angle amplification”. Indeed, such bosonic amplification of polaritons has been now adopted by the whole scientific community leading to demonstrations of exciting and exotic phenomena, such as condensation, superfluidity, soliton propagation, polariton lasing, and topological photonics. Simultaneously, it has enabled numerous applications in areas of neuromorphic and quantum computing, ultrafast switching, and single photon gating.

**Q3: During your fellowship at Cambridge University’s Cavendish Laboratory, what significant optical projects or research did you work on?**

A3: The renowned English poet Alexander Pope commemorated the outstanding English physicist Isaac Newton with an epitaph that reads:

Nature and nature’s laws lay hid in night; God said, “Let Newton be”, and all was light.

Isaac Newton, while in Cambridge University, made significant contributions to the field of optics. Newton’s most famous work on optics is his book *Opticks*, first published in 1704.

I am honored to follow in Newton’s footsteps and received the prestigious Leverhulme Visiting Professor Fellowship in 2014. This award allowed me to spend a year with Professor Jeremy Baumberg’s group at the Cavendish Laboratory, University of Cambridge. At that time, in my home University of Crete, we had just developed the ultrahigh quality Molecular Beam Epitaxy (MBE) grown semiconductor microcavity structures, which could support the propagation of polaritons in the plane of microcavities over hundreds of micrometers. This development enables a plethora of new experiments where polariton condensation could occur at large distances from the laser excitation spots, where a reservoir of excitons is created. The spatial separation of the polariton condensate from the noisy exciton reservoir allows for observations of ultrahigh temporal coherences as well as single-mode operation of these lasers, with their coherence surpassing that of widely used He-Ne lasers, opening the way for their potential use in applications requiring long-coherence lasers.

Furthermore, it leads to the spontaneous magnetization and appearance of spin-polarized polariton condensation. We are able to create large arrays of spin-polarized polariton condensates with controlled ferromagnetic or anti-ferromagnetic interactions, enabling the implementation of Ising Hamiltonians capable of solving practical optimization problems. Giant polaritonic nonlinearities, due to their strong excitonic constituent interaction, allow for electrical and optical switching of condensate spin state with optical pulses containing only a few photons or sub-femtojoule electrical pulses.

**Figure:**
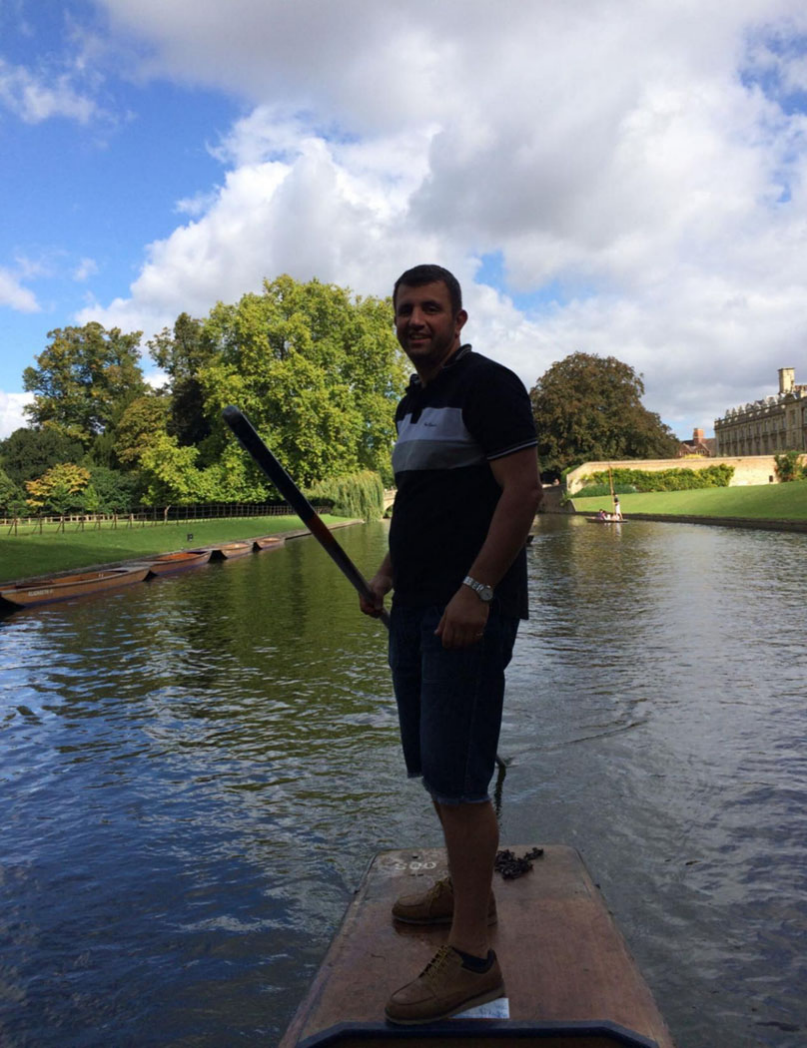
Prof. Pavlos Savvidis punting on the River Cam

**Q4: Could you elaborate on the potential applications of the technology discussed in your paper, “A GaAs polariton light-emitting diode operating near room temperature”**^1^ **published in the world-renowned journal** ***Nature*, particularly in innovative lighting solutions or optical communication?**

A4: When exciton-polaritons were first observed, it was a common perception that they would be stable only at very low temperatures and densities. Most modern semiconductor light-emitting diodes and lasers operate at near room temperature and employ high electrical current densities flowing through the diode, and polaritons face significant challenges due to the unfavorable conditions wherein their component excitons have a tendency to separate into an electron-hole plasma. Counterintuitively, our results published in *Nature* show that microcavity light-emitting diode LED based on technologically mature GaAs semiconductors can sustain polaritons emission even at near room temperature and under electrical current injection.

This achievement is made possible by the dressing of excitons with photons within a microcavity, leading to the inhibition of their mutual scattering and the subsequent prevention of their disintegration into electron-hole pairs. Follow-up experiments conducted by other research teams have demonstrated the potential for utilizing electrical injection techniques to showcase polariton lasing effects. This innovative type of lasing mechanism operates without the traditional dependence on light amplification via population inversion. Instead, it hinges on stimulated scattering and polariton condensation into a macroscopic ground state, facilitating the generation of coherent laser-like light emissions. These devices exhibit condensation or lasing thresholds that are two orders of magnitude lower than traditional lasers, making them suitable for applications requiring ultra-low power consumption. This is a major breakthrough in recent years in the research of semiconductor devices, where the previous advancements have predominantly focused on reducing size and enhancing efficiency.

**Figure:**
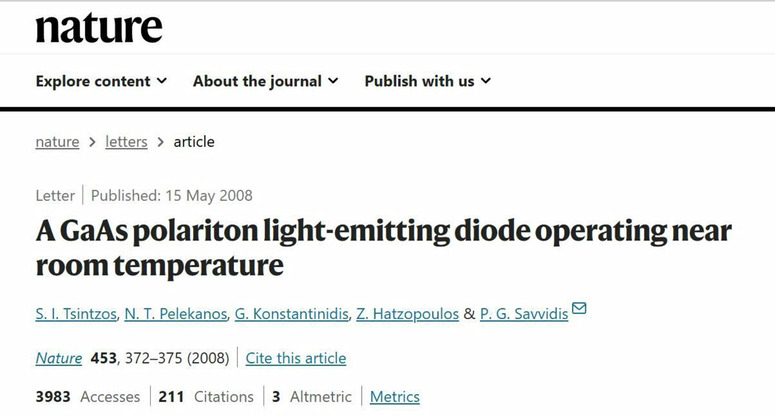
Prof. Pavlos Savvidis’ research paper published in *Nature*^1^

**Q5: One of your collaborative papers entitled “Coupling Quantum Tunneling with Cavity Photons”**^2^ **was published in the prestigious journal** ***Science*. Could you provide insights into how this research might influence photonics and quantum technologies?**

A5: Quantum tunneling devices leverage the quantum mechanical phenomenon of tunneling, allowing particles to pass through barriers that would be insurmountable according to classical physics. Examples of devices operating on this principle include Bloch Oscillators and Quantum Cascade Lasers (QCLs), which enable light emission in the mid-infrared to terahertz range.

In this work, we introduce electron tunneling to exciton systems in asymmetric quantum wells for the first time, combining it with photon dressing in a microcavity. This approach generates substantial out-of-plane electric dipole moments for polaritons, leading to significantly stronger interactions compared to their non-dipolar counterparts. These strong interactions are crucial for achieving significant nonlinear effects at the single polariton level. Owing to their enhanced interaction strengths, dipolaritons require lower power to achieve nonlinear effects, making them more practical for low-power photonic devices, such as single-photon transistors and quantum information processing systems. Additionally, the electric dipole moment of dipolaritons can be controlled by an external electric field, allowing for tunable interaction strengths and nonlinearities. This tunability provides flexibility in designing and optimizing photonic devices for specific applications. Consequently, such systems are expected to serve as an attractive platform for realizing quantum-correlated polaritons, with applications in polariton logic networks and polariton blockade.

**Figure:**
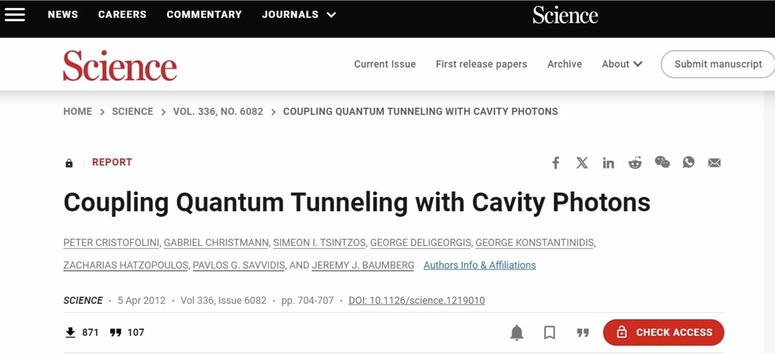
Prof. Pavlos Savvidis’ collaborative paper published in *Science*^2^

**Q6: Why do you decide to move to Hangzhou City in China to continue your research in optics? What kind of impression have the city and its people left on you?**

A6: Nearly a decade ago, my first experience of China began with a trip to Hangzhou to attend an international conference on light-matter interaction in semiconductors. During this event, we presented for the first time our work on polariton LEDs at room temperature, which had been just published in *Nature*. I was warmly greeted at the train station by my former PhD student, Tinge Gao, now an assistant professor in Tianjin University, who introduced me to the city. From that first moment, I could immediately feel the warmth, hospitality, and special reception reserved for visitors. While wandering around the scenic West Lake, I stumbled upon a statue of Marco Polo, one of the first Europeans to reach China, whose accurate and unique accounts of trade and life in 13th-century China provided invaluable insights into the region.

Now that I have moved to Hangzhou City and established a cutting-edge experimental laboratory specializing in optics, my journey mirrors that of Marco Polo, who was captivated by the region’s rich culture and technological marvels. While Polo’s explorations were driven by a quest for knowledge and trade, my mission is rooted in advancing scientific frontiers and fostering global cooperation. We both exemplify the enduring spirit of exploration and discovery, though our pursuits diverge in nature—one navigating ancient trade routes and the other probing the mysteries of light and physics within a contemporary research framework. Our parallel stories highlight Hangzhou’s timeless allure as a nexus of intellectual and cultural exchange.

**Figure:**
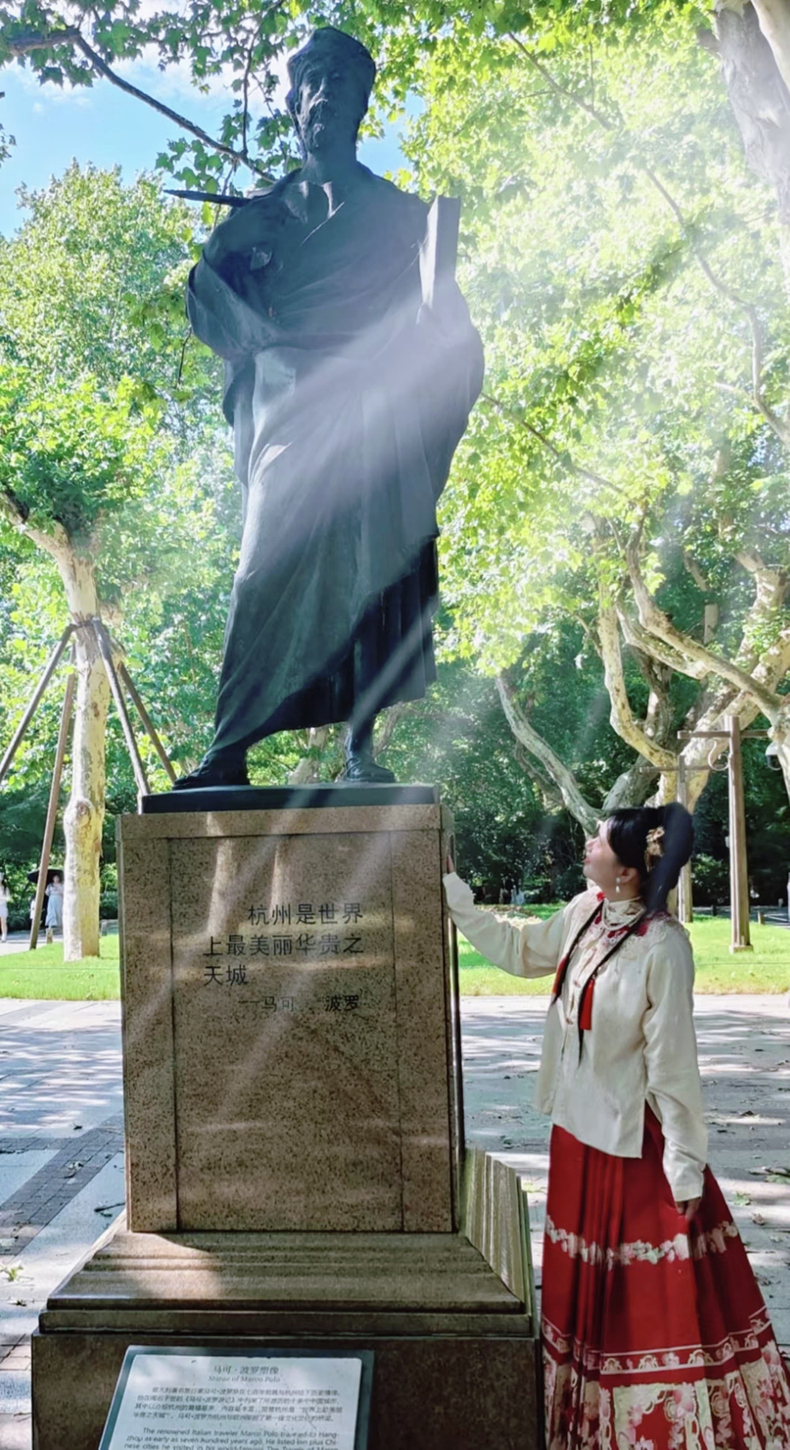
*Light* Special Correspondent standing beside Marco Polo’s statue in Hangzhou City

**Q7: Since joining Westlake University in China, how have you advanced your research on optical experiments, and what are the key objectives you aim to achieve?**

A7: Westlake University is a thriving new research and education center. I am excited to embark on a promising and captivating journey in this young university. Over the past decade, the field of polaritonics has witnessed a series of exciting developments that underscore the enormous potential of these systems for various advanced applications. At our newly established laboratories in Westlake University, one major research direction focuses on the development of a novel quantum computing platform that utilizes polariton-condensate qubits involving quantized circular currents of polaritons. This system relies on the formation of vortices in superfluids, where the phase accumulation around a supercurrent loop can only take discrete values due to the quantization of circulation. Similar principles govern the operation of superconducting flux or phase qubits involving superconducting loops interrupted by Josephson junctions.

Our progress has been strongly driven by recent achievements in attaining ultralong coherence times in polaritonic condensates within trapped geometries. This extended coherence is crucial for quantum information processing and simulation, ensuring that quantum states can be maintained over longer periods, thereby enhancing the reliability and precision of quantum computations and simulations. Additionally, polaritonic systems are easily addressable, significantly simplifying their integration into quantum devices. This ease of addressing stems from their hybrid light-matter nature, allowing for manipulation through both optical and electronic means. This dual-control capability provides a versatile toolkit for designing and implementing complex quantum simulations.

Our research also focuses on the ability to create and manipulate various quantum states within polaritonic condensates, including the formation of vortex states, solitons, and other non-trivial topological excitations. These states are essential for exploring novel quantum phenomena and developing new quantum technologies. The scalability of polaritonic systems further enhances their appeal as quantum simulation platforms.

We are also pioneering novel technologies for generating arbitrary trapping potentials and manipulating polariton condensates via high-precision He ion implantation techniques, which allow nanometer-scale patterning. Such advances in fabrication techniques enable the creation of large arrays of polaritonic condensates with precise control over their interactions. This scalability is critical for simulating larger, more complex quantum systems, which can provide new insights into novel physical phenomena.

**Figure:**
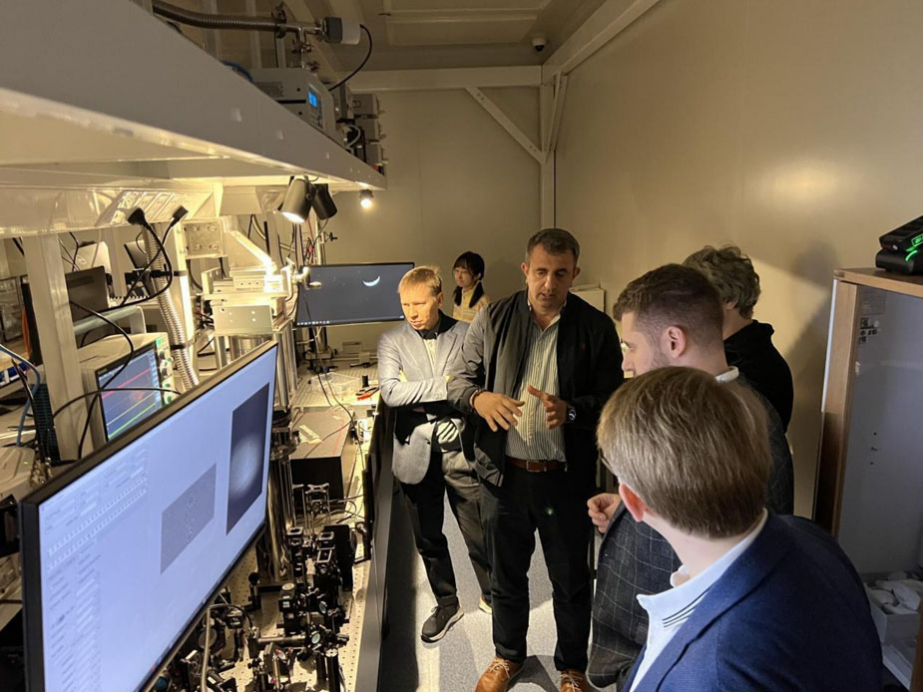
Prof. Pavlos Savvidis’s Quantum Optoelectronics Laboratory in Westlake University

**Q8: You used to be a Professor at the Department of Materials Science and Technology of University of Crete, Greece, but now is a Professor at the School of Science of Westlake University, China. From Greece to China, what roles do you think these two ancient civilizations played, currently play, and will play in the development of physics?**

A8: Both Greek and Chinese civilizations have made significant contributions to the advancement of physics, reflecting their deep-rooted commitment to scientific inquiry and education. The ancient Greeks, including philosophers like Aristotle, Archimedes, and Euclid, established the foundational principles of mechanics, optics, and hydrostatics, fostering a spirit of inquiry that shaped Western scientific thought and laid the groundwork for later scientific revolutions. Their emphasis on empirical observation and logical reasoning became the cornerstones of the scientific method. Similarly, Chinese scholars, such as Mozi, Zhang Heng, and Shen Kuo, demonstrate the sophistication of Eastern scientific endeavors. Mozi, an ancient Chinese philosopher and scientist living in the Warring States period, is particularly well-known for his studies in optics and optical instruments. Mozi proposed 8 principles of optics including pinhole imaging, which involves allowing light to pass through a small hole to form an inverted and magnified image on the opposite side. His theories laid the foundation for subsequent research in optics and the development of optical instruments, exerting a significant influence on scientists and engineers in later periods. Zhang Heng’s invention of the seismoscope and Shen Kuo’s detailed observations on magnetism and astronomy also exemplify the innovative spirit of Chinese science.

In both cultures, the value placed on education is paramount, with a strong emphasis on nurturing young minds. In Greece, the tradition of rigorous academic training dates back to the Academy of Plato and the Lyceum of Aristotle, where critical thinking and philosophical debate were encouraged, influencing countless generations of scholars. China, with its centuries-old Confucian emphasis on learning and meritocracy, has long prioritized education, evident in the highly competitive imperial examination system that selected government officials based on their knowledge and intellectual abilities.

Today, both countries continue to invest heavily in education, recognizing that fostering a new generation of thinkers is essential for future advancements in physics and beyond. In Greece, modern education systems emphasize a strong foundation in mathematics and science, encouraging students to pursue careers in research and technology. China, on the other hand, has rapidly expanded its educational infrastructure, with a focus on STEM (science, technology, engineering, and mathematics) disciplines, producing a large number of graduates who contribute to the global scientific community. This shared prioritization of education ensures that both Greek and Chinese societies continue to build on their rich legacies of scientific achievement, driving innovation and discovery in the 21st century.

The establishment of the China-Greece Belt-and-Road Joint Laboratory on Cultural Heritage Conservation Technology in Beijing marks a significant milestone in the collaboration between Greece and China, uniting two ancient civilizations in the preservation and study of their rich cultural heritage. It fosters a unique partnership where Greek and Chinese experts work side by side, sharing knowledge and techniques in archaeology, and conservation with an emphasis on optical methods applied to the preservation of cultural heritage. The laboratory serves as a hub for joint research projects, exhibitions, and educational programs, highlighting the commonalities between these two storied cultures and their contributions to human civilization. By combining Greece’s profound experience in classical antiquities with China’s advanced technological capabilities, the laboratory aims to develop innovative methods for preserving artifacts and promoting cultural exchange. This collaboration not only deepens the understanding of each other’s history and heritage but also strengthens the bonds between the two communities, demonstrating a shared commitment to the common good. Through their cooperative efforts, Greece and China showcase the power of international collaboration in safeguarding cultural legacies for future generations.

**Figure:**
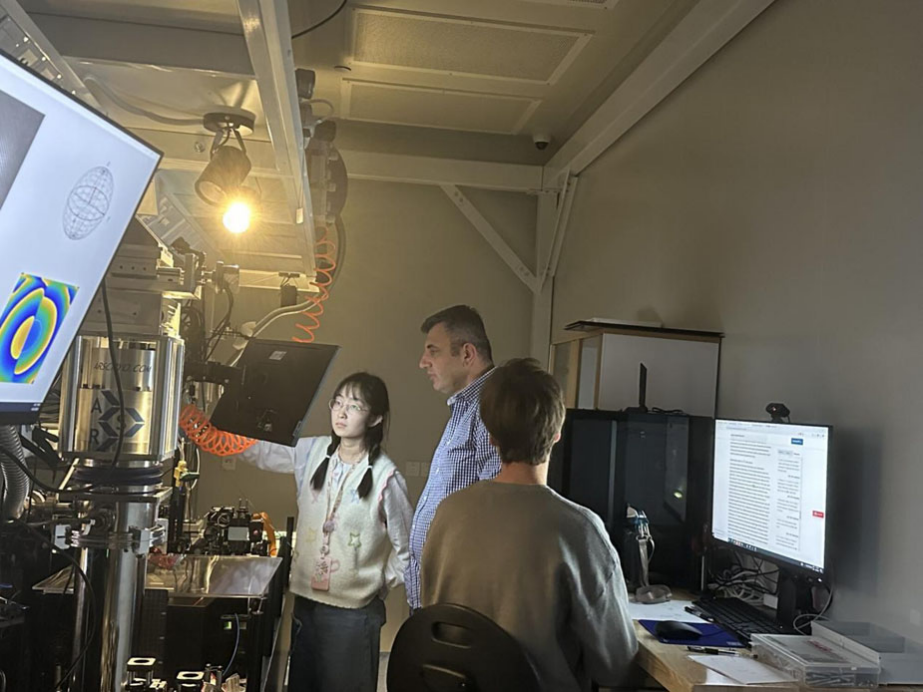
Prof. Pavlos Savvidis supervising his Chinese and international students in Westlake University

**Q9: At Westlake Coffee Bar, you once compared making espresso to conducting lab experiments. How does brewing or drinking coffee influence your research methods and scientific philosophy?**

A9: For many, drinking coffee is just a means to stay awake and energized. For others, it’s the pleasant aroma and taste, along with the opportunity to connect with others. For an experimental physicist, it’s also about the process, which, believe me, is quite sophisticated. Like any good experiment, it involves the perfect combination of multiple factors that must be carefully controlled to achieve an excellent result.

For me, making coffee has become a way to impart the importance of a meticulous experimental approach and attention to detail, while simultaneously providing the satisfaction of enjoying a great outcome. This is exactly how a well-planned and carefully executed experiment feels.

The speed at which a scientist rejects wrong ideas and approaches, ultimately determines how quickly they move towards solving a problem or uncovering the truth. This ability to swiftly discard ineffective methods or erroneous concepts is crucial in navigating the complex and often convoluted path of scientific discovery. This persistent, diligent, tentative, and adaptive approach is the hallmark of successful scientific practice and is instrumental in the achievement of groundbreaking discoveries.

**Figure:**
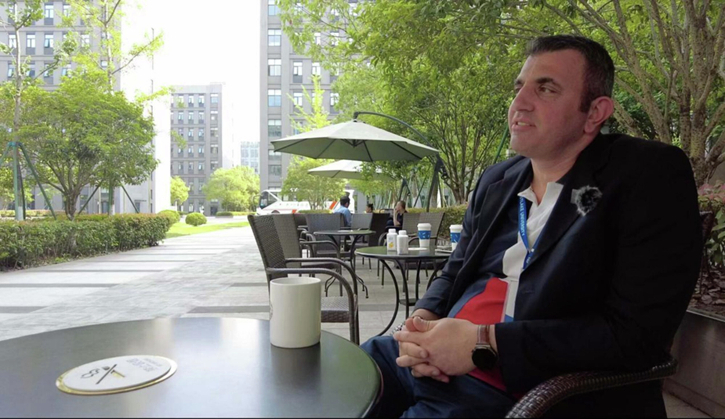
Prof. Pavlos Savvidis drinking coffee on Westlake Yunqi campus

**Q10: How do you balance work and leisure during your time in China?**

A10: Adapting from a Mediterranean lifestyle to life in China is certainly not an easy task. However, discovering new gastronomic journeys has been truly fascinating for me while adapting to these new ways of life. During this time, I have uncovered hidden cooking skills, which I strongly link to my experimental background. My kitchen has become my secret experimental laboratory, where new cooking methods and techniques are constantly being explored.

This culinary experimentation has rekindled my enthusiasm for hands-on, creative processes, helping me sustain a sense of exploration and innovation despite the increasing demands of research and administrative responsibilities. It serves as a personal reminder of the joy of discovery and the satisfaction of creating something new, much like the experimental work that initially drew me to science.

**Figure:**
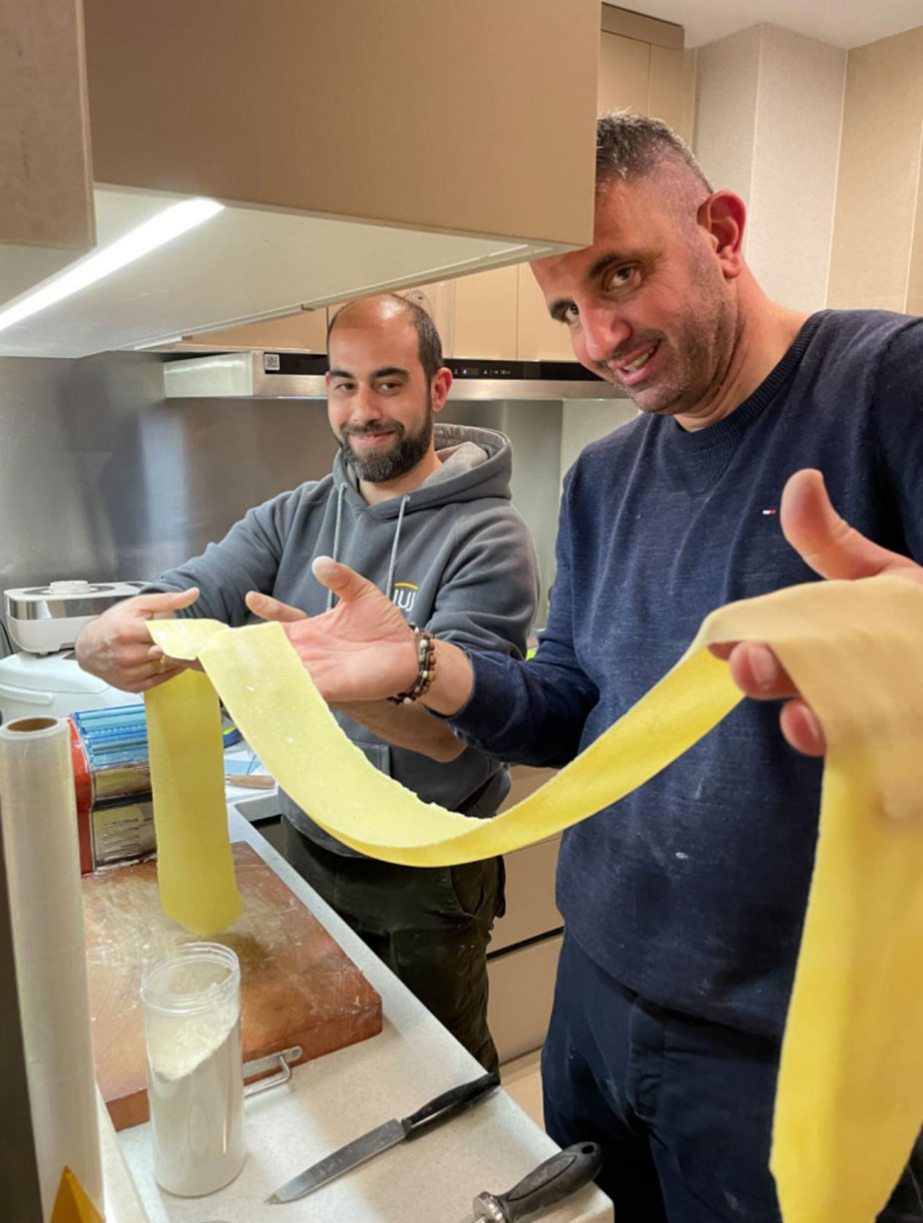
Prof. Pavlos Savvidis making fresh homemade pasta for ravioli

**Q11: What qualities do you believe define a great scientist? What advice would you give to young students and aspiring scientists?**

A11: Having spent many years in research, I have come to realize that a successful scientific career is defined by a diverse bouquet of qualities, including a solid educational background, persistence, endurance, intuition, and the often-overlooked abilities of human interaction. In the modern scientific environment, many interesting ideas are born from interactions with other researchers, who bring different perspectives and expertise, leading to fundamentally novel ideas and approaches. These skills are usually not taught in universities and must be developed by researchers during their careers. A great scientist and a team leader should strive with his or her presence, skills, and leadership on the frontline, stimulating, motivating, and energizing the research team.

Perhaps the greatest satisfaction in my scientific career comes from witnessing the true transformation of the young scientists who join my group. When they find motivation, unlock their true potential, and ultimately succeed in their careers, it is immensely rewarding. This personal transformation often surpasses the satisfaction of publishing a top-quality scientific result in a major journal. My advice to students is of course to explore the ideas that we discuss and plan during our meetings, but also as experimentalists to be constantly on the lookout for something unusual and unexpected, because this is exactly how new big discoveries are often made.

**Figure:**
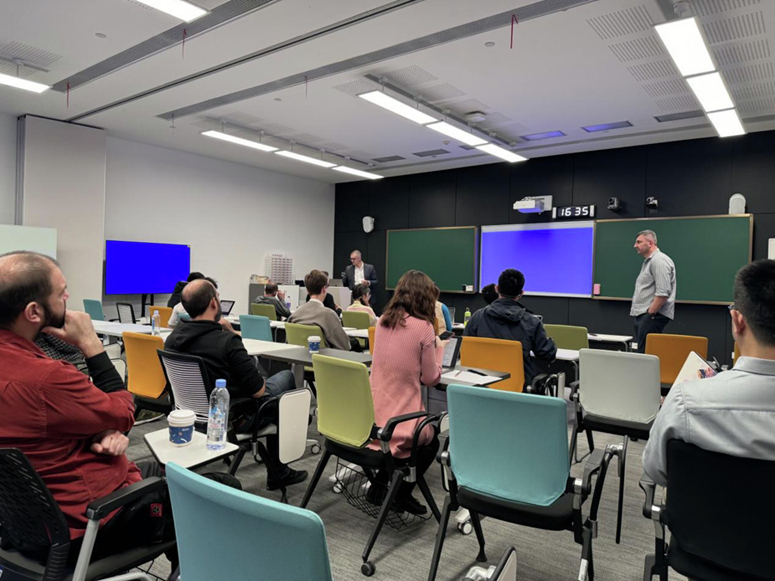
Prof. Pavlos Savvidis hosting an invited lecture in Westlake University

**Q12: How do you perceive the advancements in optical scientific research in China over the past decade? What potential opportunities do you see for future collaborations between Chinese and international researchers?**

A12: Clearly, over the past decade, China has invested heavily in the development of centers of excellence for optics, leveraging its vast human potential both from within its borders and from talented young researchers returning from abroad with expertise in cutting-edge technologies. Young principal investigators are encouraged to undertake independent research initiatives, supported by substantial investments in scientific infrastructure and instrumentation. Unlike the global trend of prioritizing the quantity of produced research, China emphasizes fundamental, open-ended research as well as addressing significant scientific and technological challenges in key interdisciplinary areas of interest such as quantum computing and quantum information science, biomedical research, and advanced materials and nanotechnology.

Moreover, an increasing number of my colleagues from around the world have visited China, drawn by the growing opportunities and the appeal of Chinese universities for international collaboration. Policies, such as the adoption of English as the viable medium of communication and instruction, have significantly increased the appeal of Chinese academic institutions to international scholars. In my group, we regularly host exchange visits from foreign researchers, providing excellent opportunities for collaborative projects. Access to state-of-the-art facilities and the wealth of expertise available at Chinese universities often serves as a crucial catalyst for initiating such collaborative initiatives.

**Figure:**
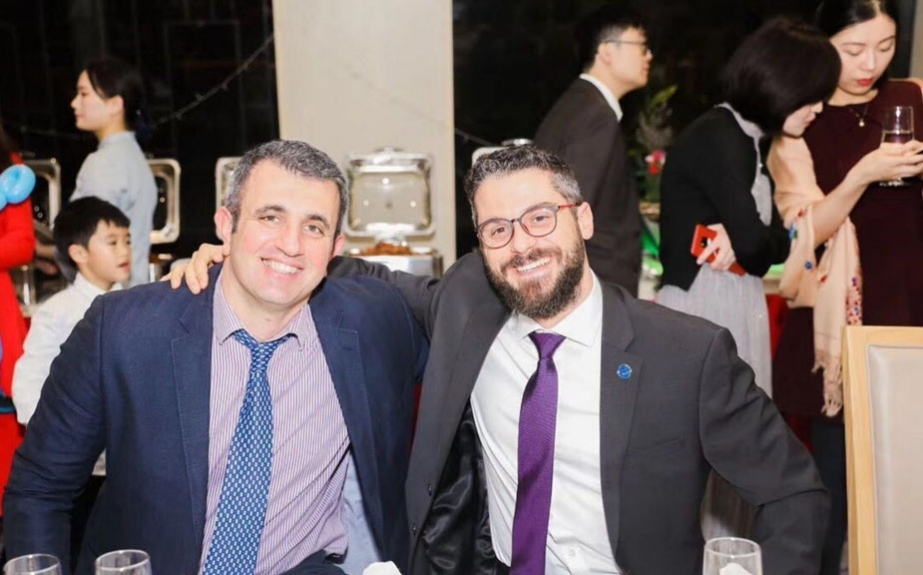
Prof. Pavlos Savvidis and another Greek Westlake University PI, Prof. Konstantinos Lagoudakis, on a boat excursion on the Qiantang River in Hangzhou City
